# Intermedin promotes vessel fusion by inducing VE‐cadherin accumulation at potential fusion sites and to achieve a dynamic balance between VE‐cadherin‐complex dissociation/reconstitution

**DOI:** 10.1002/mco2.9

**Published:** 2020-06-09

**Authors:** Lingmiao Kong, Fei Xiao, Lijun Wang, Min Li, Denian Wang, Zhongxue Feng, Luping Huang, Yong'gang Wei, Hongyu Li, Fei Liu, Yan Kang, Xuelian Liao, Wei Zhang

**Affiliations:** ^1^ Department of Critical Care Medicine State Key Laboratory of Biotherapy and Cancer Center West China Hospital Sichuan University and Collaborative Innovation Center of Biotherapy Chengdu China; ^2^ Department of Intensive Care Unit of Gynecology and Obstetrics West China Second University Hospital Sichuan University Chengdu China; ^3^ Department of Liver Surgery West China Hospital Sichuan University Chengdu China; ^4^ Liver Transplantation Center Beijing Friendship Hospital Capital Medical University Chengdu China; ^5^ Department of Critical Care Medicine West China Hospital Sichuan University Chengdu China

**Keywords:** angiogenesis, intermedin (IMD), Rab4, Rab11, vascular development, vascular endothelial cadherin (VEC), vessel fusion

## Abstract

To create a closed vascular system, angiogenic sprouts must meet and connect in a process called vessel fusion, which is a prerequisite for establishment of proper blood flow in nascent vessels. However, the molecular machinery underlying this process remains largely unknown. Herein, we report that intermedin (IMD), a calcitonin family member, promotes vessel fusion by inducing endothelial cells (ECs) to enter a “ready‐to‐anchor” state. IMD promotes vascular endothelial cadherin (VEC) accumulation at the potential fusion site to facilitate anchoring of approaching vessels to each other. Simultaneously, IMD fine‐tunes VEC activity to achieve a dynamic balance between VEC complex dissociation and reconstitution in order to widen the anastomotic point. IMD induces persistent VEC phosphorylation. Internalized phospho‐VEC preferentially binds to Rab4 and Rab11, which facilitate VEC vesicle recycling back to the cell‐cell contact for reconstruction of the VEC complex. This novel mechanism may explain how neovessels contact and fuse to adjacent vessels to create a closed vascular system.

## 1 INTRODUCTION

Angiogenesis, a process by which the primary vessel network is expanded via sprouting of new vessels from the preexisting vasculature, plays important roles in many physiological and pathological processes, particularly in wound healing and cancer.^1^, ^2^, ^3^ To effectively provide blood supply, the angiogenic vessels must be organized into an elaborate hierarchical system of appropriate density.^3^, ^4^ Although the majority of cancerous tumors are highly vascularized, the vessels within the tumors are abnormal with regard to almost all aspects of their structure and function.^5^, ^6^ The tumor vasculature contains numerous angiogenic sprouts that form dead ends and do not fuse to the established circulatory network, which severely impairs blood perfusion.

To create a closed vascular system, angiogenic sprouts must meet and connect in a process called vessel anastomosis or vessel fusion, which is a prerequisite for blood flow through the vessels.^3^, ^4^ Unfortunately, few studies have focused on the process of vessel fusion. According to Fantin et al, macrophages act as cellular chaperones for vascular anastomosis.^7^ However, the molecular machinery that stimulates and regulates vessel fusion remains largely unknown.^3^, ^4^ Our previous work^8^, ^9^, ^10^ revealed that intermedin (IMD; also named adrenomedullin 2 [ADM2]), a member of the calcitonin family,^11^ normalizes the tumor vasculature and effectively improves tumor blood supply. IMD dramatically remodels the vasculature morphology into a hierarchical architecture that is well organized with relatively fewer sprouts,^9^ larger lumens,^8^ and more anastomosed vessels.^10^ Because IMD significantly reduces vessel density while increasing the number of anastomotic vessels,^9^, ^10^ we hypothesize that IMD may be an important molecule that stimulates vessel fusion, thus contributing to vessel normalization and blood perfusion improvement.

## 2 RESULTS

### 2.1 IMD induces hierarchical and functional vasculature development, indicating that it may contribute to vessel fusion

**FIGURE 1:**
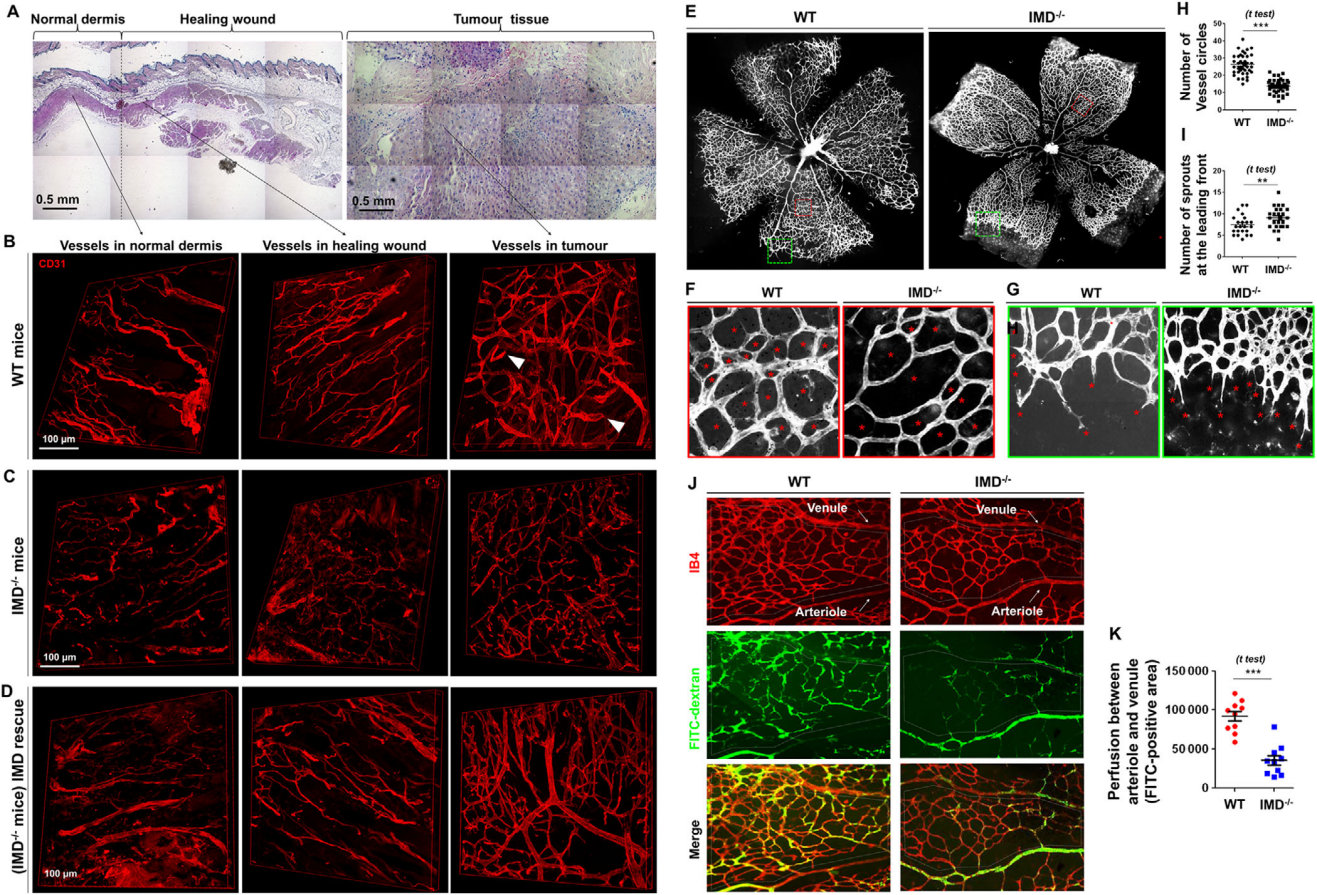
IMD promotes vessel fusion in vitro and in vivo. A, H&E histological analysis of the normal dermis, healing wound, and tumor tissue (subcutaneously inoculated Lewis lung cancer) from C57/BL6 mice. B‐D, Immunofluorescent analysis (stained for CD31) of the vascular system in normal dermis, healing wounds, and tumor tissues from WT, IMD^−/−^, and IMD^−/−^ mice rescued by IMD peptide injection. The white arrows in panel (B) indicate the representative blind‐ended vessels. E‐I, Retinal vasculature of WT and IMD^−/−^ mice (stained with IB4). F, Asterisks indicate the vessel circles between the retinal arteriole and venule. G, Asterisks indicate the vessel sprouts at the leading front of retinal vasculature. H and I, Quantification of vessel circles and sprout tips using 20 randomly chosen fields from five samples. J, WT or IMD^−/−^ mice were injected with FITC‐dextran to mark the perfused vessels. Retinas were stained with IB4. Dash line circles the area between arteriole and venule. K, The effective perfusion (FITC‐positive area) between arteriole and venule was quantified by the software Image‐Pro Plus v5.0.2.9 (similarly hereinafter) using 10 randomly chosen fields. The data were presented as scatter plots with mean ± SEM. Significance was assessed by unpaired, two‐tailed parametric *t* test with Welch's correction (H, I, and K). IMD^−/−^: intermedin knockout; FITC: fluoresceine isothiocyanate; WT: wildtype

As shown in Figures 1A and 1B, in normal tissues and physiological angiogenic (ie, wound healing) zones, vessels sprouted from the existing vasculature and connected to adjacent vessels to form closed vascular systems allowing blood flow. However, in tissues with pathological angiogenesis, particularly in tumors (Figures 1A and 1B; right panels), the sprouting vessels were often blind‐ended and did not fuse to the circulatory networks. The decreased vessel connectivity led to stagnant or completely dead ends, even in highly vascularized areas (Figure 1B). Compared with wild‐type (WT) mice, IMD‐knockout (KO) (*IMD* ^−/−^) mice exhibited significantly more blind‐ended vessels in both the physiological and tumor vasculatures (Figure 1C). To confirm the specificity of the vascular phenotype observed in *IMD* ^−/−^ mice, a rescue experiment was performed. Injection of the mature IMD peptide into *IMD* ^−/−^ mice increased vessel connectivity under both physiological and pathological conditions, enhancing the hierarchical nature of the vasculature (Figure 1D).

Impaired vessel hierarchical structure was also observed in the developing retinal vasculature in *IMD* ^−/−^ mice (Figure 1E). We counted the vessel rings between the arteriole and venule to indirectly assess successful vessel anastomosis (Figure 1F). The number of vessel rings was approximately 48% lower in *IMD* ^−/−^ mice than in WT mice (Figure 1H). Because increases in vessel ring numbers may be due to increased vessel branching, we counted the tips of vessels that sprouted from the leading front of the retinal vasculature. The number of vessel sprouts in *IMD* ^−/−^ mice was significantly greater than that in WT mice (Figure 1G and 1I). This finding is consistent with our previous observation that IMD restricts rather than promotes vessel branching.^9^ Our previous work has revealed that IMD increases tumor blood perfusion, whereas IMD blockade decreases tumor blood perfusion.^10^ Herein, in the retinal model, we found that effective perfusion of the capillaries between the arteriole and venule was severely impaired in the *IMD* ^−/−^ mice (Figures 1J and 1K), consistent with our previous observation.

### 2.2 IMD promotes vessel fusion in in vitro and in vivo models

**FIGURE 2:**
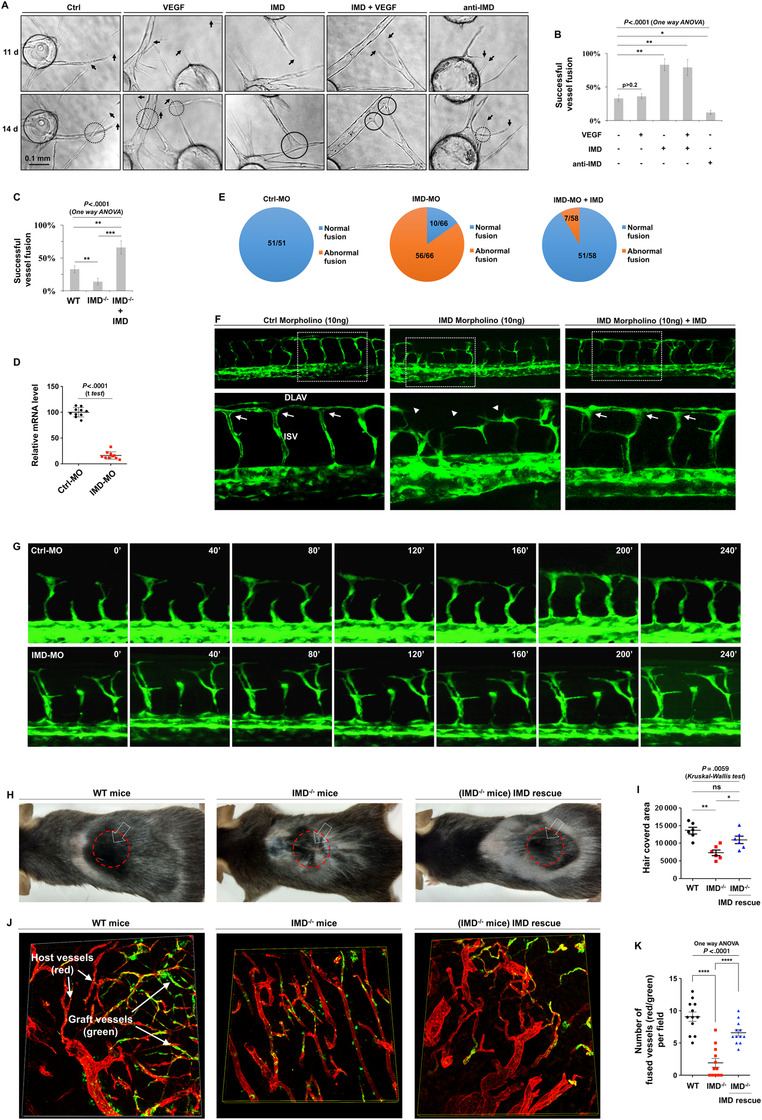
The dynamic process of IMD‐induced vessel fusion. A, Fibrin beads assay: VEGF (50 ng/mL), IMD (2 µM), or anti‐IMD (25 µg/mL; same doses hereinafter) were added at day 1. Images in the same field were captured at day 11 and day 14. B, The successful fusion rates were calculated using 10 randomly chosen vessels from two independent experiments. C, Fibrin beads assay was performed using endothelial cells (ECs) isolated from WT, KO, and IMD‐rescued KO mice. The successful fusion rates were calculated using 10 randomly chosen vessels from two independent experiments. D, Zebrafishes treated with Ctrl‐MO or IMD‐MO (10 ng). The mRNA level of IMD was measured by real‐time RT‐PCR (n = 10). E, The number of normal/abnormal fusions in Ctrl‐MO, IMD‐MO, or IMD‐MO + IMD peptide rescued group. F, Representative images of zebrafishes treated with Ctrl‐MO, IMD‐MO, or IMD‐MO rescued by IMD peptide. Arrows indicate the normal ISV/DLAM fusion. Arrow heads indicate the abnormal ISV/DLAV fusion. G, The time‐lapse microphotography with a 40‐min interval showing the normal or IMD‐MO‐induced abnormal ISV/DLAV fusion process. H, WT or IMD^−/−^ mice received skin transplant surgery. The hollow arrow pointed the skin graft (1 cm × 1 cm), and the red circle marked the hair regrowth area. I, The hair regrowth area in the transplant site was quantified (n = 6). J, Skin grafts from the GFP‐transgenic mice (green) were transplanted onto the backs of WT or *IMD* ^−/−^ mice. After the grafts begun to regrow hair, the grafts and surrounding tissues were entirely removed and stained with CD31 (red) to display all blood vessels. The blood vessels from GFP^+^‐grafts show green/red overlapped fluorescence, whereas the host vessels show only red fluorescence. K, The connection between red and green/red double‐positive vessels was quantified, which indicates that the host and graft vessels are successfully fused (n = 12). The data were presented as columns with mean ± SD. Significance was assessed by one‐way ANOVA (Kruskal‐Wallis test) followed by nonparametric Dunn's post hoc analysis. anti‐IMD: antibody to intermedin; Ctrl: control; FITC: fluoresceine isothiocyanate; IMD^−/−^: intermedin knockout; MO: morpholino; VEGF: vascular endothelial growth factor; WT: wildtype

Histological images can provide only snapshots of vessel growth at certain time points. To observe the vessel fusion process more dynamically, we established a three‐dimensional in vitro angiogenic model (fibrin bead assay)^12^ to monitor the continuous growth of vascular sprouts and the approach, contact, and fusion of the sprouts with other vessels (Figure 2A). Vascular endothelial growth factor (VEGF) is believed to be the most important growth factor during angiogenesis. However, VEGF alone was unable to induce the sprouting vessels to form the fine connections necessary for establishment of a hierarchical vessel system. In the vehicle‐ and VEGF‐treated groups, the adjacent vessels approached reciprocally, but most of them crossed over without connecting or fusing (Figure 2A). On the other hand, IMD dramatically promoted the contact of approaching vessels and the formation of fine connections in the presence or absence of VEGF. In addition, treatment with a monoclonal antibody targeting IMD (anti‐IMD) inhibited this effect (Figure 2A). Compared with the control, IMD significantly increased the rate of successful vessel fusion by approximately 2.5‐fold, and anti‐IMD decreased the rate by approximately threefold (Figure 2B). IMD is expressed in blood vessels and endothelial cells (ECs).^10^ Thus, we examined fusion in ECs isolated from *IMD* ^−/−^ mice and found that the successful fusion rate was significantly lower in *IMD* ^−/−^ mouse ECs than in normal WT mouse ECs; in addition, the reduction was reversed by exogenous IMD administration (Figure 2C).

The experimental accessibility and optical clarity of zebrafish embryos make these animals good models for observation of the dynamic vessel fusion process in vivo.^13^ In normal embryos, the intersomitic vessels (ISVs) sprout from the dorsal aorta and then fuse with the dorsal longitudinal anastomosing vessels (DLAVs).^14^ We used morpholinos (IMD‐MO and Ctrl‐MO as a control; sequences provided in Section 4) to knock down IMD expression in Tg (flk1:eGFP) transgenic zebrafish. The gene knockdown (KD) efficiency was verified by real‐time RT‐PCR (Figure 2D). For a gene rescue experiment, the mature zebrafish IMD (zIMD) peptide was synthesized and added to the fish water to reach a final concentration of 2 µM. The vessel fusion between ISVs and DLAVs was severely impaired in the IMD‐MO‐injected zebrafish; 56 of 66 embryos showed abnormal anastomosis. The abnormal vessel fusion in IMD‐MO‐injected zebrafish was significantly alleviated by zIMD administration, after which only five of 59 embryos showed abnormal anastomosis. No abnormalities were observed in the Ctrl‐MO‐treated zebrafish (Figures 2E and 2F). Time‐lapse imaging showed the progression of vessel fusion in the Ctrl‐MO‐ and IMD‐MO‐treated zebrafish (Figure 2G).

Given the ability of IMD to promote vessel anastomosis, we hypothesized that IMD may help transplant grafts regain function, as vascular anastomosis between the graft and host is critical for restoration of blood supply. To test this hypothesis, we performed skin transplant surgery using WT and *IMD* ^−/−^ mice. Compared with WT mice, *IMD* ^−/−^ mice exhibited significant delays in skin graft function restoration (as indicated by hair regrowth). IMD injection reversed this effect (Figures 2H and 2I). To directly observe anastomosis between the graft vessels and the host vessels, we acquired skin grafts from GFP‐transgenic mice (labeled with green fluorescence) and transplanted them onto the backs of WT and *IMD* ^−/−^ mice. After the grafts began to regrow hair, the grafts and surrounding tissues were removed entirely and stained with CD31 (labeled with red fluorescence) to display all blood vessels. In theory, with this method, only the blood vessels from the skin graft will show both green and red fluorescence, whereas the blood vessels from the host will show only red fluorescence. Therefore, connections between red and green blood vessels indicate that the host and graft vessels are successfully fused. Upon counting the successfully anastomosed blood vessels, we found that IMD KO significantly impaired the process of vessel fusion, whereas IMD peptide supplementation alleviated this defect (Figures 2J and 2K). According to the above results, IMD may be a key molecule in vessel fusion and blood supply restoration.

### 2.3 IMD enhances the anchoring ability of adjacent vessels to facilitate successful vessel fusion

**FIGURE 3:**
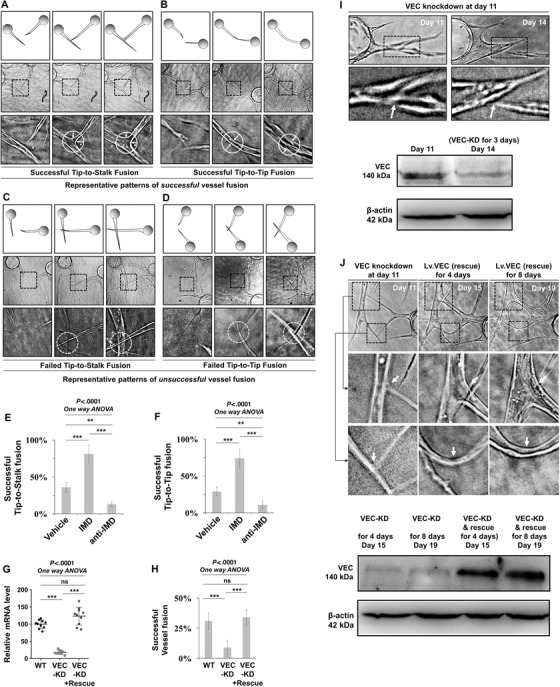
The IMD‐induced vessel fusion is VEC dependent. A and B, Fibrin beads assay (described in Section 4) was performed to observe the process of vessel fusion in vitro. Representative patterns of successful and unsuccessful tip‐to‐stalk and tip‐to‐tip vessel fusion. The dash‐line box outlined the partial enlarged view of the area of vessel fusion. The dash‐line circles outlined the contact and fusion sites. The white arrows indicated the widening of the fusion site. C and D, Representative patterns of successful and unsuccessful tip‐to‐stalk and tip‐to‐tip vessel fusion. The dash‐line box outlined the partial enlarged view, and the dash‐line circles indicated that the approaching vessels contact but do not anchor with each other, resulting in unsuccessful vessel fusion. E and F, The successful tip‐to‐stalk and tip‐to‐tip fusion rates (treated with vehicle, IMD, or anti‐IMD) were calculated using 10 randomly chosen vessels from two independent experiments. G, The VEC‐knockdown and VEC‐knockdown and rescue efficiency was tested using real‐time RT‐PCR (n = 10). H, The successful fusion rates (VEC‐knockdown or VEC‐knockdown and rescue) were calculated using 10 randomly chosen vessels from two independent experiments. I, Representative images showing the impaired vessel fusion site after VEC‐knockdown for 3 days (upper panels). The VEC expression level was assessed by western blot (WB) assay (lower panels). Arrows indicated the detachment of the already established anastomotic point. J, Representative images showed that the VEC rescue (VEC‐knockdown followed by Lv.VEC transfection) repaired the anastomotic point and leads to successful vessel fusion (upper panels). VEC expression level was assessed by WB (lower panels). KD: knockdown; VEC: vascular endothelial cadherin

We then investigated how IMD facilitates the process of vessel fusion. Two major patterns of vessel fusion, tip‐to‐tip fusion and tip‐to‐stalk fusion, were observed (Figures 3A and 3B). During successful vessel fusion, vessel tips approached and anchored to adjacent vessel stalks or vessel tips and formed fine anastomoses with connected hollow lumens (Figures 3A and 3B); however, during unsuccessful fusion, although adjacent vessels approached and contacted reciprocally, they were unable to anchor to each other and instead crossed over each other (Figures 3C and 3D). Statistical analysis showed that IMD markedly increased the successful fusion rates of both the tip‐to‐stalk and tip‐to‐tip patterns and indicated that these effects could be inhibited by anti‐IMD (Figures 3E and 3F).

IMD has been reported to regulate vascular endothelial cadherin (VEC), an endothelial‐specific transmembrane component of adherens junctions,^10^, ^15^, ^16^ which are the major junctional structures at EC cell‐cell contact zones.^17^, ^18^ VEC is recruited to the fusion point when an ISV fuses to a DLAV in zebrafish,^13^ and stable vessel anastomosis requires full expression of VEC. A partial reduction in VEC expression compromises the capacity for establishment of successful reciprocal contacts.^19^ Thus, VEC‐medicated EC‐EC adhesion may be crucial for successful vessel fusion. We hypothesized that IMD may promote the formation of the VEC complex between reciprocally approaching ECs, thereby enhancing the anchoring ability of the adjacent vessels and facilitating successful vessel fusion. We performed an RNA interference and rescue experiment to test this hypothesis. VEC transcription levels were assessed by real‐time RT‐PCR (Figure 3G). KD of VEC via targeting of the 3′‐UTR of VEC mRNA significantly impeded the ability of IMD to induce vessel fusion (Figure 3H).

We next investigated whether VEC KD would affect already established anastomotic points. Cells in fibrin beads were transfected with siRNA‐VEC at day 11. The VEC KD efficiency was determined by western blot (WB) assay (Figure 3I, lower panels). We found that the already established vessel fusion sites dissociated after VEC KD for 3 days (Figure 3I, upper panels). To determine whether VEC expression would repair this disruption, we performed a VEC rescue experiment by transfecting the cells in this system with Lv.VEC after VEC KD at day 11. The VEC expression levels at day 15 and day 19 (after VEC KD or VEC KD rescue for 4 days and 8 days, respectively) were evaluated by WB assay (Figure 3J, lower panels). We found that VEC rescue reestablished the impaired fusion sites of adjacent vessels (Figure 3J, upper panels) and facilitated successful vessel fusion.

### 2.4 IMD induces ECs to enter a “ready‐to‐anchor” state by fine‐tuning the behavior of VEC to achieve a dynamic balance between VEC complex dissociation and reconstitution

**FIGURE 4:**
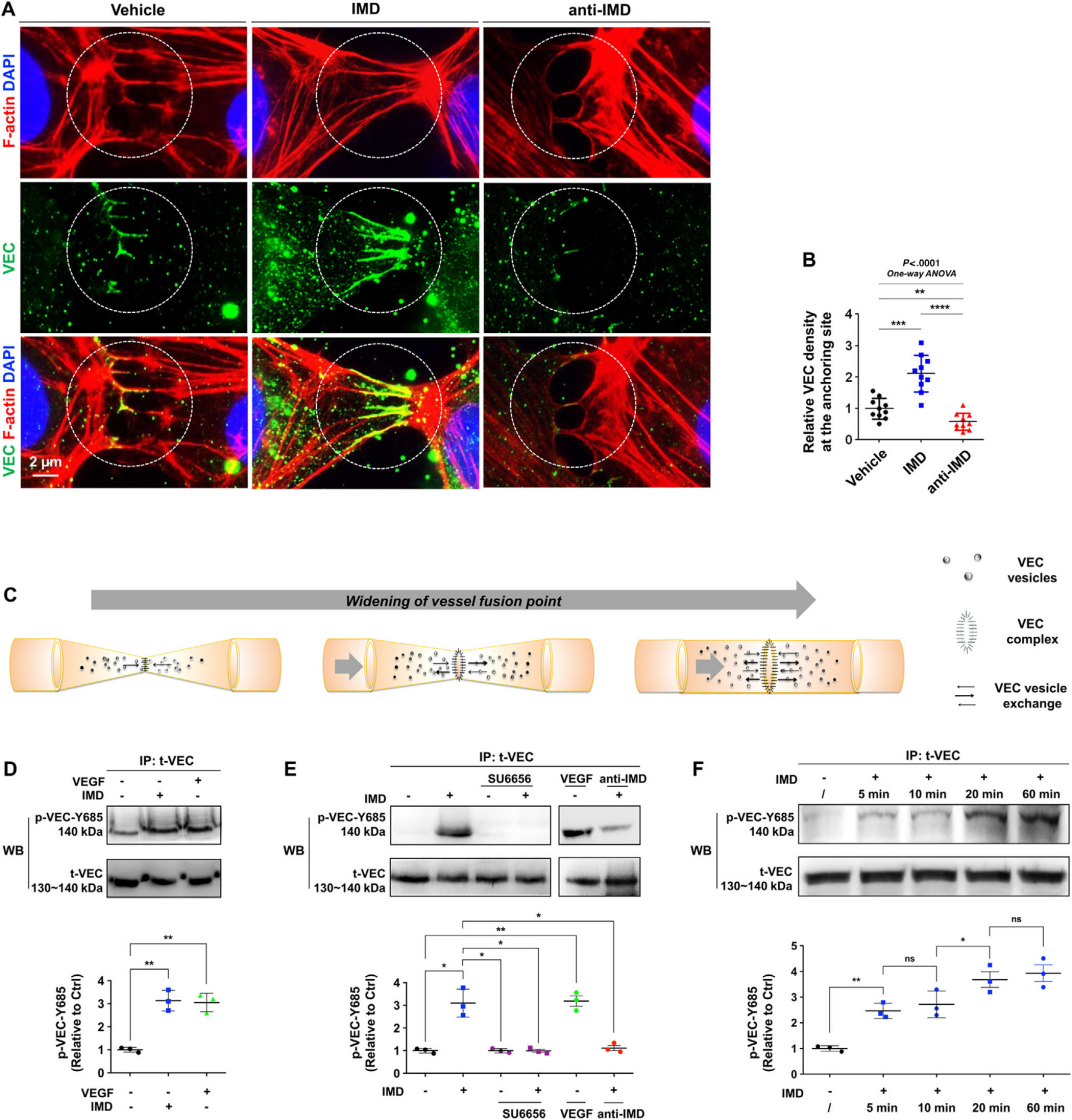
IMD relocates VEC to the potential contact point and induces VEC phosphorylation. A, Double staining for filopodia (Phalloidin, red) and VEC (green) showed the newly formed VEC complexes between close but not tightly touched ECs. B, The staining density of VEC relative to phalloidin was quantified, as described in Section 4. C, Schematic: the widening of vessel fusion point. First, adjacent vessels contact and anchor to each other; second, the anastomotic point expand continuously for the interconnection of adjacent lumenized vessels. The whole process is accompanied by VEC vesicle transportation and redistribution. D, HUVECs treated with IMD or VEGF were immunoprecipitated (IP) by anti‐total‐VEC and immunoblotted (IB) by anti‐pY685‐VEC. E, The HUVECs were pretreated with SU6656 (5 µM for 30 min), followed by IMD treatment for 10 min were subjected to the IP‐IB assay; the cells treated by VEGF and anti‐IMD were tested as controls. F, Samples from HUVECs treated by IMD for 5, 10, 20, and 60 min were subjected to the IP‐IB assay using anti‐total‐VEC and anti‐pY685‐VEC. D and F, The levels of p‐VEC (referred to t‐VEC) were presented relative to control; n = 3. Data were presented as scatter plots with mean ± SD. Significance was assessed by one‐way ANOVA (Kruskal‐Wallis test) followed by nonparametric Dunn's post hoc analysis (B) or unpaired, two‐tailed parametric *t* test with Welch's correction (D‐F). IP: immunoprecipitation; p‐VEC: phosphorylated VEC; t‐VEC: total‐VEC; VEC: vascular endothelial cadherin; WB: western blot

IMD has been reported to stabilize endothelial junctions^10^, ^15^, ^20^, ^21^; however, it is unclear whether IMD facilitates the formation of new VEC complexes between ECs that are adjacent but not yet fully contacting each other, which is a prerequisite for successful vessel anchoring. Herein, we investigated whether IMD affects VEC distribution at cell‐cell contact areas. We found that IMD treatment significantly increased the accumulation of VEC in filopodial bridges that connected adjacent ECs, whereas anti‐IMD treatment markedly decreased VEC accumulation (Figures 4A and 4B). These results indicate that IMD may facilitate vesicular VEC transport to potential contact points. Thus, IMD may help VEC translocate to potential contact points, therefore inducing ECs to enter a ready‐to‐anchor state. This process may help adjacent vessels make good contacts and form VEC complexes, resulting in an increased chance of successful vessel fusion.

We then realized that good contact between adjacent ECs is necessary but not sufficient to achieve successful vessel fusion. As shown in Figure 3A‐D, successful vessel fusion requires three steps: contact, anchoring, and widening of the anastomotic point. After the approaching sprouts anchor to each other, the anastomotic point must be expanded to enable interconnection of adjacent lumenized vessels (Figure 4C). If IMD only strengthens the VEC complex at the cell‐cell contact point and prevents the dissociation of this complex, the adjacent ECs will be firmly fixed to the anastomotic point, which will restrict the widening of the anastomotic site and lead to unsuccessful vessel fusion. Thus, the dynamic dissociation and reconstitution of the VEC complex are indispensable processes for successful vessel fusion.

Autophosphorylation of VEC leads to VEC complex dissociation and VEC endocytosis.^18^ Because IMD has been reported to stabilize the endothelial barrier,^10^, ^15^, ^20^, ^21^ IMD might affect VEC phosphorylation. WB and immunoprecipitation (IP) assays revealed that IMD increased VEC phosphorylation at Y685 in HUVECs, similar to the effect of VEGF (Figures 4D and S1A). However, IMD did not phosphorylate VEC at Y658 or Y731 as VEGF did (Figure S1B). The cytoplasmic domain of VEC contains nine tyrosine residues that represent potential phosphorylation sites. According to Wallez, VEC is a direct substrate for Src kinase, and Y685 is the unique site phosphorylated by active Src.^22^ Our recent work has revealed that IMD induces Src phosphorylation in vitro and in vivo and that the specific Src inhibitor SU6656 blocks constitutive and IMD‐induced Src phosphorylation.^8^ Herein, the IP/WB assay revealed that SU6656 inhibited IMD‐induced VEC phosphorylation (Figure 4E). As positive and negative controls, VEGF and anti‐IMD induced and inhibited the phosphorylation of VEC, respectively (Figure 4E). According to the results, IMD may activate VEC via the Src signaling cascade. An IP time course assay revealed that 5 min of IMD treatment induced VEC phosphorylation; this effect became gradually stronger with longer treatment durations (Figure 4F). Tyrosine phosphorylation usually peaks approximately 5‐10 min after stimulation and then declines rapidly. Thus, IMD may exert a persistent effect on VEC activity.

**FIGURE 5:**
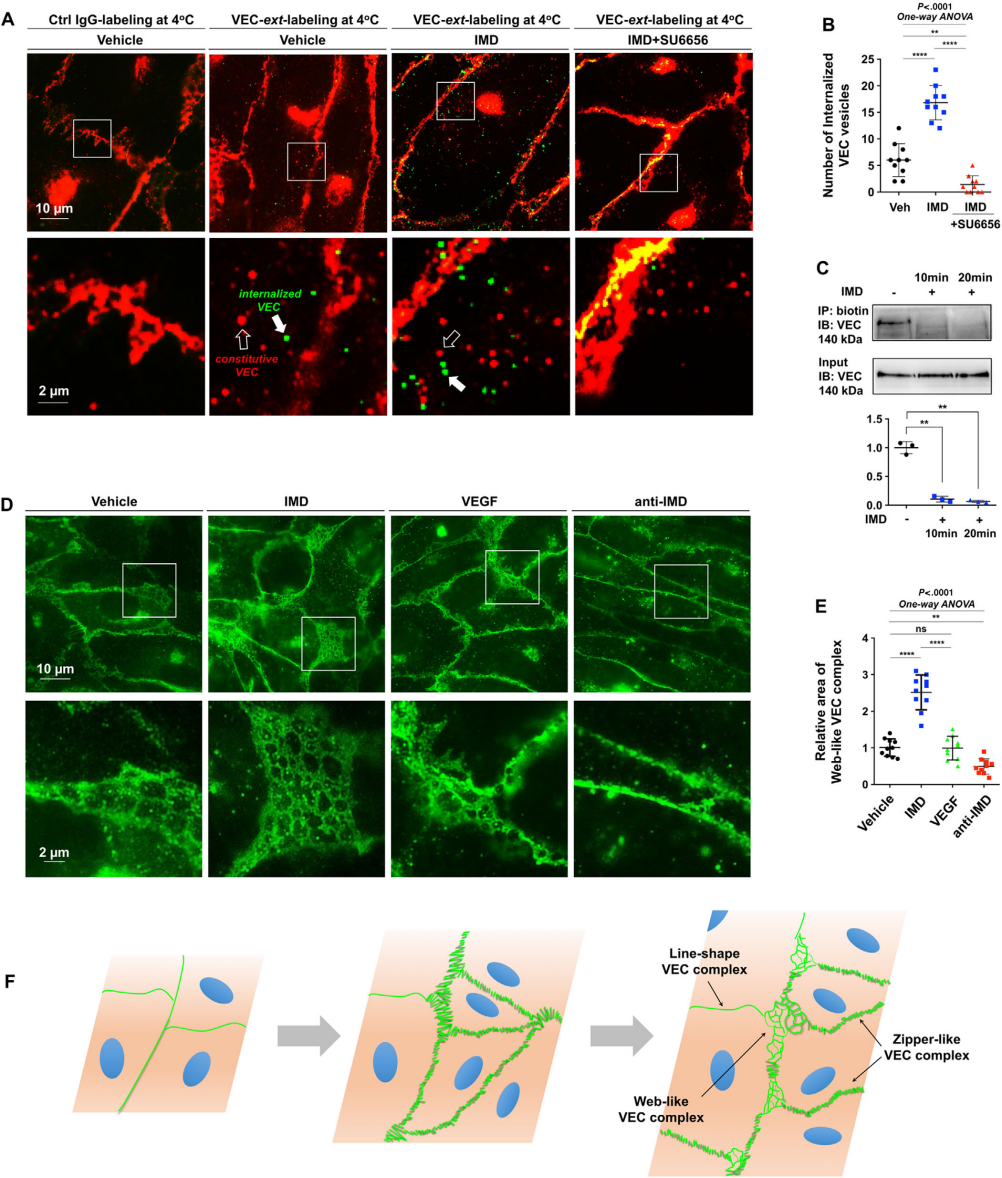
IMD fine‐tunes the behavior of VEC to achieve a dynamic balance between VEC complex dissociation and reconstitution. A, HUVECs were labeled with anti‐VEC‐*ext* (recognizing the extracellular domain of VEC), and stimulated with vehicle and IMD (with or without pre‐treatment of SU6656), followed by detection using secondary antibody (green). After successful staining was confirmed, the cells were incubated with the anti‐VEC‐*int* (recognizing the intracellular domain of VEC; red). The white box outlined the partial enlarged view of the area near the cell‐cell contact. The hollow arrows pointed the constitutive VEC vesicles labeled by *anti‐VEC‐int*, and the solid arrows pointed the internalized VEC vesicles labeled by anti‐VEC‐*ext*. B, The number of internalized VEC was calculated. C, Surface‐biotinylation assay was performed, and the level of biotin‐labeled VEC on the cell surface was detected (n = 3). D, The representative images showing the web‐like VEC complex in HUVEC monolayer treated by vehicle, IMD, VEGF, or anti‐IMD. E, The area covered by web‐like structure was quantified (n = 10). F, The expansion of anastomotic point was needed to enlarge the vessel lumen via EC proliferation. The newly proliferated ECs from the pre‐existing ECs will inevitably lead to the “old” VEC complex dissociation and the “new” VEC complex reconstitution, resulting in coexistence of three types of VEC complex: line‐shape, zipper‐like, and web‐like structure. All quantifications use 10 randomly chosen fields from two independent experiments. Data were presented as scatter plots with mean ± SEM. Significance was assessed by one‐way ANOVA (Kruskal‐Wallis test) followed by nonparametric Dunn's post hoc analysis. IP: immunoprecipitation; VEC‐ext: antibody recognizing the extracellular domain of VEC; VEC‐int: antibody recognizing the intracellular domain of VEC; WB: western blot

VEC internalization is considered a result of VEC phosphorylation. Herein, we used two VEC antibodies, one recognizing the extracellular domain of VEC (named VEC‐*ext*) and another recognizing the intracellular domain of VEC (named VEC‐*int*). HUVECs were labeled with VEC‐*ext* or nonspecific IgG for 30 min at 4°C, stimulated with vehicle and IMD (with or without pretreatment with SU6656) for 20 min at 37°C, and then stained using an Alexa Fluor 488‐conjugated secondary antibody (green). After successful staining was confirmed under a microscope, the cells were incubated with the VEC‐*int* antibody and subsequently stained with Alexa Fluor 568 (red). We calculated the numbers of internalized VEC vesicles and found that IMD significantly increased them; furthermore, SU6656 nearly completely inhibited this effect (Figures 5A and 5B). We then performed a surface biotinylation assay to confirm this effect. The results showed that IMD stimulation significantly reduced the levels of biotin‐labeled VEC on the cell surface (Figure 5C), indicating that the increased numbers of VEC vesicles in the cytoplasm were indeed coming from the cell surface.

VEC internalization is considered a result of VEC complex dissociation. Thus, although IMD promoted the formation of VEC complexes, it concurrently induced VEC complex destabilization and VEC internalization. According to these results, IMD may fine‐tune the behavior of VEC to achieve a dynamic balance between VEC complex dissociation and reconstitution, which is a prerequisite for anastomotic point expansion, as demonstrated in Figure 4C.

We next examined the EC monolayer because the ECs that constitute blood vessel walls are in contact with each other, similar to confluent cultured ECs. Interestingly, other than the linear and zipper‐like VEC complexes, we observed unique web‐like structures between the ECs in close contact with one another (Figure 5D). The presence of these unique structures suggested the occurrence of a dynamic process of simultaneous VEC complex dissociation and reconstruction. Our recent study revealed that IMD enlarges the vessel lumen by inducing the proliferation of quiescent ECs.^8^ Because the ECs that constitute blood vessels wall are in close contact with each other, the proliferation of new ECs from preexisting ECs and the consequent enlargement of the vessel lumen inevitably lead to the dissociation of “old” VEC complexes followed by the reconstitution of “new” VEC complexes, as demonstrated in Figure 5F. This is unlikely to be a unique phenomenon caused by a certain growth factor; rather, it is likely a naturally occurring phenomenon. Stimulation with IMD significantly increased the frequency and the areas covered by these web‐like structures, whereas treatment with anti‐IMD markedly decreased these parameters. VEGF, although considered to be the most important growth factor in angiogenesis, showed no obvious effect on the formation of the structures (Figures 5D and 5E).

### 2.5 IMD promotes VEC transportation via both the Rab4 and Rab11 recycling routes

**FIGURE 6:**
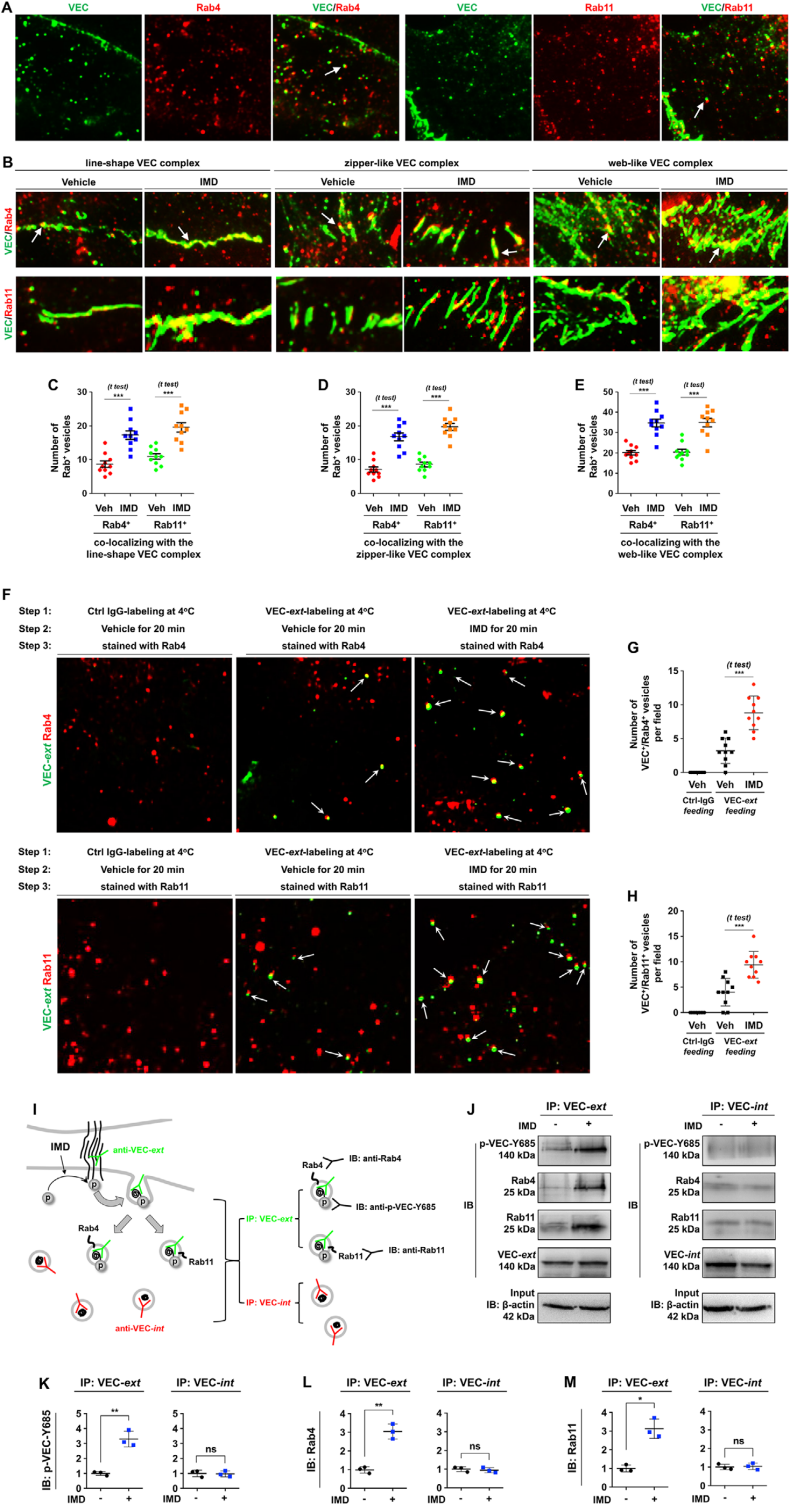
IMD promotes VEC vesicle transportation via Rab4 and Rab11. A, HUVECs were double stained with VEC/Rab4 or VEC/Rab11. Arrows indicate the co‐localization of VEC/Rab. (B) The accumulation of Rab4^+^ or Rab11^+^ vesicles on three types of VEC complexes: line‐shape, zipper‐like, and web‐like structure. Arrows indicate Rab4^+^ or Rab11^+^ vesicles co‐localization with VEC complex. C‐E, Quantification of Rab4^+^ or Rab11^+^ vesicles accumulated on the line‐shape (C), zipper‐like (D), and web‐like VEC complex (E). F, The HUVECs were labeled with VEC‐*ext* (an antibody recognizing extracellular domain of VEC, green) or nonspecific IgG, and incubated with vehicle or IMD‐stimulated VEC internalization. After successful staining was confirmed, the cells were double stained for Rab4 or Rab11 (red). G and H, The number of VEC^+^/Rab4^+^ and VEC^+^/Rab11^+^ vesicles were counted (n = 10). I, The diagram shows how to detect the interaction of phospho‐VEC and Rab4/Rab11: HUVECs were labeled with VEC‐*ext* and stimulated with IMD to induce VEC phosphorylation. The cells lysates were collected and immunoprecipitated with VEC‐*ext* and VEC‐*int*, respectively. The precipitated proteins were then immunoblotted using anti‐Rab4 or anti‐Rab11 antibodies. If the phosphorylated VEC preferentially binds to Rab4/Rab11, the anti‐VEC‐*ext*‐immunoprecipitated protein complex will be recognized by anti‐Rab4 and anti‐Rab11 antibodies, but the anti‐VEC‐*int*‐immunoprecipitated protein complex will not. J, The IP‐IB assay detected the interaction between phospho‐VEC and Rab4/Rab11. K‐M, The density of the band (referred to total‐VEC) was presented relative to that of the control. The mean level in the control group was set to 1.0; n = 3. Significance was assessed by unpaired, two‐tailed parametric *t* test with Welch's correction. IB: immunoblotting; IP: immunoprecipitation; VEC: vascular endothelial cadherin; Veh: vehicle; VEC‐ext: antibody recognizing the extracellular domain of VEC; VEC‐int: antibody recognizing the intracellular domain of VEC

As shown in Figure 5B‐G, IMD potently induced VEC phosphorylation and subsequent VEC internalization. These findings raised the following question: after VEC is internalized into the cytoplasm, how is it transported back to the cell membrane? The small GTPases Rab4 and Rab11 facilitate rapid and slow canonical recycling routes for membrane proteins, respectively.^23^ Using multiple immunofluorescence staining, we found that VEC endosomes colocalized with either Rab4 or Rab11 in the cytoplasm of resting ECs (Figure 6A). This result indicates the existence of a constitutive recycling process for VEC. Treatment with IMD did not affect the expression of either Rab4 or Rab11 in HUVECs (Figure S2). However, IMD significantly increased the number of Rab4^+^ and Rab11^+^ vesicles that accumulated precisely on the linear, zipper‐like, and web‐like VEC complexes (Figure 6B‐E). According to these results, IMD may induce Rab4‐ and Rab11‐carrying VEC endosomes to return to cell‐cell contact points to rebuild VEC complexes.

To test whether IMD can directly promote binding of internalized VEC to Rab4/Rab11, thus entering the membrane‐protein recycling process, we designed a three‐step experiment. In Step 1, cells were labeled with VEC‐*ext* (an antibody recognizing the extracellular domain of VEC). In Step 2, the labeled cells were incubated with vehicle or IMD for 20 min to stimulate VEC internalization and stained with an Alexa Fluor® 488‐conjugated (green) secondary antibody for 30 min for detection of internalized VEC. In Step 3, after successful staining was confirmed, the cells were double‐stained with anti‐Rab4 or anti‐Rab11 antibodies and an Alexa Fluor® 568‐conjugated (red) secondary antibody. The results showed that IMD significantly promoted binding of internalized VEC to Rab4/Rab11 (Figure 6F‐H). These findings raised another question: why does internalized VEC tend to bind to Rab4/Rab11? Interestingly, we noticed that IMD treatment did not induce merely transient VEC phosphorylation; rather, it induced persistent VEC phosphorylation, which became gradually stronger with longer treatment durations (Figure 5D). This is not a common phenomenon. It is well known that tyrosine phosphorylation usually peaks for 5‐10 min after stimulation and then declines rapidly. Thus, we sought to investigate the role of persistent phosphorylation, hypothesizing that such phosphorylation helps VEC bind to Rab4/Rab11.

We designed an experiment to test this hypothesis. After cells were labeled with VEC‐*ext* and stimulated with IMD to induce VEC phosphorylation and internalization, the cell lysates were collected and subjected to IP with VEC‐*ext* and VEC‐*int* separately. As demonstrated above, IMD induced persistent VEC phosphorylation; thus, at 20 min after IMD stimulation, internalized VEC should still be phosphorylated and able to be recognized by an anti‐VEC‐Y685 antibody. In addition, if phosphorylated VEC preferentially binds to Rab4/Rab11, the anti‐VEC‐*ext*‐immunoprecipitated protein complex should be able to be recognized by anti‐Rab4 and anti‐Rab11 antibodies, but the anti‐VEC‐*int*‐immunoprecipitated protein complex should not. A diagram of the experimental design is shown in Figure 6I. The results confirmed our hypothesis that upon IMD stimulation, internalized phospho‐VEC preferentially bound to Rab4/Rab11, but nonphosphorylated VEC did not (Figure 6J‐M). Taken together, the experimental findings described above show that IMD directly promotes VEC internalization from the cell surface by inducing persistent VEC‐Y685 phosphorylation. The internalized phospho‐VEC preferentially binds to Rab4 and Rab11, which can facilitate VEC vesicle transport in the cytoplasm and recycling back to cell‐cell contact areas.

### 2.6 The active form of Rab4 or Rab11 is required for VEC transportation but may not be necessary for VEC cargo binding

As mentioned above, immunofluorescence analysis of cell slides can provide only a snapshot of the cell status at a certain time point. To acquire more direct evidence of whether VEC is recycled via Rab4 or Rab11 in living cells, HUVECs were transfected with lentiviruses expressing VEC (red) and either Rab4 or Rab11 (green) and observed under time‐lapse microphotography at 15‐s intervals. The VEC vesicles were bound to either Rab4 (Figure 6F) or Rab11 (Figure 6G), indicating that VEC endosomes were concurrently recycled through either a Rab4‐ or Rab11‐dependent route. The continuous microphotography time lapse revealed that over certain time periods, one or several endosomes in a field could travel long distances across the cytoplasm, whereas others usually vibrated in a Brownian manner (Videos 1 and 2 in the Supporting Information). One Rab4‐bound VEC vesicle took approximately 8 min to travel from the cytoplasm to the cell border, and a Rab11‐bound VEC vesicle took approximately 18 min to travel across a similar distance (Figures 6F and 6G; Videos 1 and 2 in the Supporting Information). IMD significantly increased the numbers, ranges, and speeds of long‐range‐moving Rab4‐ and Rab11‐bound VEC vesicles (Figures 6H and 6I; Videos 3 and 4 in the Supporting Information). KD of Rab4 or Rab11 partially impeded the ability of IMD to promote VEC transportation (Figures 6J‐L and S3). To exclude possible off‐target effects, cells were transfected with small hairpin RNA (shRNA) targeting the 3′‐UTR of either Rab4 or Rab11; the KD was subsequently reversed by transfection of Rab4^WT^ or Rab11^WT^ constructs that did not contain the 3′‐UTR, respectively. The constitutive and IMD‐induced VEC movement were both restored (Figure 6J‐L). When Rab4 and Rab11 were silenced at the same time, the movement of VEC endosomes was nearly blocked, and IMD lost its ability to promote VEC movement (Figure 6J‐L). According to these results, IMD may promote VEC transportation via both the Rab4 and Rab11 recycling routes.

**FIGURE 7:**
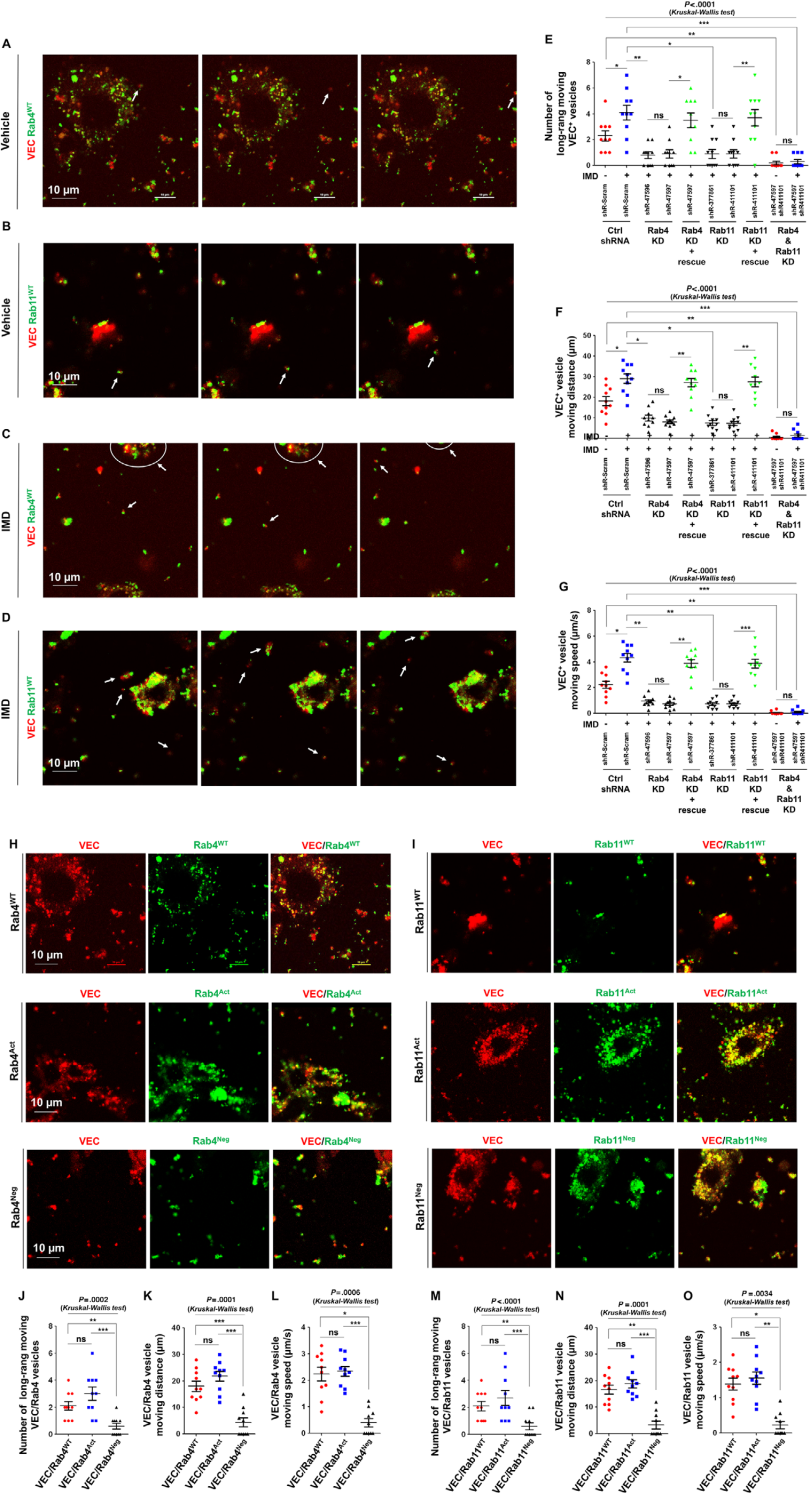
The active form of Rab4 or Rab11 is required for VEC transportation. A‐D, HUVECs were co‐transfected with lentivirus expressing VEC/Rab4^WT^ or VEC/Rab11^WT^, treated without IMD (A and B) or with IMD (C and D), and observed under continuous microphotography with a 15‐s interval. Arrows indicate the long‐range moving VEC^+^/Rab4^WT+^ or VEC^+^/Rab11^WT+^ vesicles. E‐G, HUVECs were transfected with shRNA to knockdown Rab4 (shR‐47596 targets ORF; shR‐47597 targets 3’‐UTR) or Rab11 (shR‐377861 targets ORF; shR‐411101 targets 3’‐UTR), and rescued by transfection of Lentiviral Rab4^WT^ or Rab11^WT^, and incubated with or without IMD. The number of long‐range moving VEC^+^ vesicles and their moving distance and speed were quantified. H and I, HUVECs were co‐transfected with Lentiviral VEC (red) and Rab4^WT^/Rab4^Act^/Rab4^Neg^ (green) or Rab11^WT^/Rab11^Act^/Rab11^Neg^ (green), respectively. J‐O, The number of long‐range moving VEC^+^/Rab^+^ vesicles and their moving distance and speed were quantified. All quantifications use 10 randomly chosen fields from two independent experiments. Data were presented as scatter plots with mean ± SEM. Significance was assessed by one‐way ANOVA (Kruskal‐Wallis test) followed by nonparametric Dunn's post hoc analysis. Act: active; IMD^−/−^: intermedin knockout; KD: knockdown; Neg: negative; shRNA: small hairpin RNA; VEC: vascular endothelial cadherin; WT: wildtype

Small GTPases, including Rab4 and Rab11, have two states: active and inactive, during which they bind GTP or GDP, respectively. To determine whether the activity status of Rab4 and/or Rab11 affects VEC transportation, GTP‐bound constitutively active mutant forms of Rab4 and Rab11 (Rab4[Q67L] and Rab11[Q70L], abbreviated as Rab4^Act^ and Rab11^Act^, respectively) and GDP‐bound dominant‐negative mutant forms (Rab4[S27N] and Rab11[S25N], abbreviated as Rab4^Neg^ and Rab11^Neg^, respectively) were constructed (labeled with green). HUVECs were co‐transfected with VEC‐expressing lentiviruses (red) and the mutant constructs. The constitutively active forms (Rab4^Act^/Rab11^Act^) bound to nearly all VEC endosomes, similar to the respective WT constructs (Figures 7A and 7B). Surprisingly, Rab4^Neg^ and Rab11^Neg^ also bound to VEC (Figures 7A and 7B); this was unexpected because the dominant‐negative mutants of Rab4 and Rab11 were assumed to be unable to bind their cargoes. Sequential microphotography revealed that the movement of VEC/Rab4^Act^ and VEC/Rab11^Act^ vesicles was slightly greater than that of VEC/Rab4^WT^ and VEC/Rab11^WT^ vesicles (Figure 7C‐H and Videos 5 and 6 in the Supporting Information). However, the movement of VEC/Rab4^Neg^ and VEC/Rab11^Neg^ vesicles was significantly decreased (Figure 7C‐H and Videos 7 and 8 in the Supporting Information). Thus, the active form of Rab4 or Rab11 is required for VEC transportation but may not be necessary for VEC cargo binding.

## 3 DISCUSSION

During vascular development and angiogenesis, vessel sprouts must meet, connect, and fuse to adjacent vessels to create a closed vascular system. The vessel fusion process is crucial in establishing a hierarchical and functional vasculature. However, the molecular mechanism underlying this process remains largely unknown. In this study, we found that an endogenous peptide, IMD, functions as a key mediator to stimulate vessel fusion by precisely regulating the behavior of VEC. According to previous studies, VEC is recruited to the EC interface during the fusion of ISVs and DLAVs,^13^, ^24^ and successful vessel fusion requires stable cell‐cell junctions that are defective in the absence of VEC.^19^ Although the importance of VEC in vascular fusion has been demonstrated, the molecular mechanism by which VEC participates in the regulation of vascular fusion remains unclear.

In this study, we found that IMD regulates the behavior of VEC in two ways. First, IMD increases VEC accumulation at the potential fusion point, thereby inducing ECs to enter a ready‐to‐anchor state that facilitates the anchoring of approaching vessels to each other via VEC complex formation. Second, IMD not only induces VEC complex dissociation and VEC endocytosis via Src‐mediated VEC‐Y685 autophosphorylation but also facilitates VEC complex rebuilding by promoting VEC endosome transport back to the cell‐cell contact point via Rab4‐ and Rab11‐mediated vesicle trafficking routes.

Our results indicated that IMD directly promoted VEC internalization from the cell surface by inducing persistent VEC‐Y685 phosphorylation. The internalized phospho‐VEC preferentially bound to Rab4 and Rab11, but nonphosphorylated VEC did not. The role of IMD in promoting VEC vesicle movement may be due to the characteristics of Rab4 and Rab11. In this process, perhaps the most important effect of IMD is promotion of VEC and Rab4/Rab11 binding; the rest of the process is mediated by Rab4 and Rab11. As the cargo vehicle molecules of fast and slow canonical recycling routes, respectively, Rab4 and Rab11 can facilitate VEC vesicle transport in the cytoplasm and recycling back to cell‐cell contact areas.

Previous studies have noted the importance of VEC‐mediated formation of stable reciprocal contact between adjacent vessels during vascular fusion.^19^ Although good contact between adjacent ECs is indeed necessary, it is not sufficient for successful vessel fusion. If the VEC complex at a cell‐cell contact point does not dissociate, the adjacent ECs will become firmly fixed at the anastomotic point, anastomotic site widening will be prohibited, and functional connection of the lumenized vessels will be impossible. Our results suggest that vascular fusion is a highly dynamic process in which IMD fine‐tunes the behavior of VEC to achieve a dynamic balance between VEC complex dissociation and reconstitution. The unique effect of IMD on VEC also ensures that when fusion sites are widened, the ECs at both ends remain connected with newly formed VEC complexes. This activity ensures that vascular leakage does not occur, which may result when the old VEC complexes dissociate.

Although vessel fusion is a crucial step in angiogenesis and vascular development, only a few studies have focused on this process. Fantin and colleagues have reported that macrophages may act as cellular chaperones for vascular anastomosis.^7^ However, vascular fusion can still occur without the presence of macrophages near vascular junctions. According to a study by Kubota and colleagues, although macrophage defects lead to decreases in the density of vascular networks at the beginning of vascular development, a relatively normal vascular system is eventually formed.^25^ In this study, we observed two patterns of vessel fusion (tip‐to‐tip and tip‐to‐stalk) in the three‐dimensional in vitro angiogenesis model (fibrin bead assay). This system contained only ECs and skin fibroblasts; it did not contain macrophages. These results suggest that macrophages may facilitate the process of vascular fusion but may be dispensable.

In the first week after birth, the retinal blood vessels in mice are still in the developmental stage and do not cover the entire retinas. After postnatal day 7 (P7), the retinal vasculature begins to mature, and some small branches regress. As reported by Fantin and colleagues,^7^ retinal vascular regression is detectable from P9 to P21. In this study, the eyes of neonatal WT and IMD‐KO C57BL/6 mice were collected at P6. At this time point, the occurrence of vascular regression is very rare. Therefore, vessel regression was unlikely to be the main cause of the reduced blood perfusion. According to the results, impaired lumen enlargement and decreased vessel fusion may have been the main causes of the reduced blood perfusion in IMD‐KO mice.

Shear stress caused by blood flow is also important for vascular remodeling.^26^, ^27^ Here, we observed successful vessel fusions with connected open lumens occurring in the absence of blood flow (Figures 2A and 3, fibrin bead assay). This result suggests that at least in the early stages of angiogenesis and vascular development when neovessels form a primitive vasculature, development of a closed vascular network may be induced by a flow‐independent mechanism. At this stage, molecular manipulation may be the primary way to regulate the vessel fusion process. In the later stage, when the primitive vessel network is remodeled into a hierarchical and functional vasculature, blood flow is established, and mechanical stress may begin to participate in the vascular reshaping process.

Taken together, our data suggest a crucial role of the IMD‐Rab4/Rab11‐VEC signaling axis in vessel fusion. To the best of our knowledge, IMD is the first endogenous molecule observed to be capable of promoting vascular fusion without the aid of other angiogenic factors or cells. IMD acts as a driver, VEC acts as an effector, and Rab4/Rab11 acts as a “cargo vehicle” to transport VEC endosomes to the areas in which EC contact is most likely to occur. Importantly, we have revealed that vessel fusion is a dynamic process in which IMD precisely controls VEC to achieve a dynamic balance between VEC complex dissociation and reconstitution. This machinery is consistent with the natural process of vascular fusion and is supported by our experimental data. Thus, a flow‐independent molecular mechanism underlying vessel fusion has been revealed. The ability of IMD to promote vessel fusion may be useful for blood supply restoration after transplantation or during wound healing and for broadening of antiangiogenic drug screening via targeting of the vascular anastomotic process.

## 4 METHODS

### 4.1 Cells and culture conditions

Lewis lung cancer cell line LL/2 was obtained from ATCC and routinely cultured in complete DMEM. HUVECs were isolated from umbilical cords and cultured in EGM‐2 (Lonza, Cat. CC‐3162). Human skin fibroblasts (HSFs) for co‐culturing with HUVECs were isolated from surgical specimens and routinely grown in complete DMEM. All cells were tested for mycoplasma contamination, and routinely culture at 37°C and 5% CO_2_.

### 4.2 Reagents

Anti‐VEC antibody (Cat. #2500, react to intracellular domain) and anti‐Rab11 (Cat. #5589) were obtained from Cell Signaling Transduction. Anti‐CD31 antibody (Cat. 550274) and anti‐VEC (Cat. 555661, react to extracellular domain) were from BD Bioscience. Anti‐VEC‐Y685 (Cat. ab119785) was from Abcam. Anti‐VEC‐Y658 (Cat. 441144), anti‐VEC‐Y731 (Cat. 441145), Alexa Fluor 594‐conjugated IB4 (Cat. I21413), and Alexa Fluor 568‐conjugated Phalloidin (Cat. A12380) were from Invitrogen. Anti‐β‐actin (sc‐47778) was from Santa Cruz. SU6656 (Cat. S9692), anti‐Rab4 (Cat. R5780), and fluoresceine isothiocyanate (FITC)‐conjugated dextran with a molecular weight of 2000 kDa (Cat. FD2000S) were from Sigma‐Aldrich. The anti‐IMD mAb (clone number: 1106CT1.2.1; isotype: IgG2b) was customized by Abgent. Lentiviral vectors expression mCherry‐VEC, eGFP‐Rab4^WT^, eGFP‐Rab4^Act^ ([Q67L] mutation), eGFP‐Rab4^Neg^ ([S27N] mutation), eGFP‐Rab11^WT^, eGFP‐Rab11^Act^ ([Q70L] mutation), and eGFP‐Rab11^Neg^ ([S25N] mutation) were customized and provided by Genechem. IMD (IMD_8‐47_, the mature form of IMD peptide, containing 40 amino acid residues) was synthesized and purchased from Shinegene. siRNA targeting ORF of VEC (A‐003641‐14, A‐003641‐15, and A‐003641‐17), siRNA targeting 3’UTR of VEC (A‐003641‐16), shRNA targeting ORF of Rab4 (Clone ID: V2LHS_47596), shRNA targeting 3’UTR of Rab4 (Clone ID: V2LHS_47597), shRNA targeting ORF of Rab11 (Clone ID: V3LHS_377860), and shRNA targeting 3’UTR of Rab11 (Clone ID: V3LHS_411101) were from Dharmacon.

### 4.3 *IMD(ADM2)* ^−/−^ mice

The *IMD(ADM2)^−/−^* mice were obtained using CRISPR/Cas9 system as described previously.^8^ In brief, two sgRNAs were designed targeting the specific locations in the third exon of *murine*‐*IMD* gene. The *IMD/Cas9* plasmids were injected into the zygotes of C57BL/6J mice. The mutation of IMD was identified by PCR in 21 of 28 mice. The PCR products were then cloned and sent to sequence. The sequencing result showed that there was a 317‐bp deletion (highlighted) in 27# mice, and coding sequence of IMD was entirely removed. No fetal death or neonatal death was found in the IMD‐KO line.

### 4.4 Zebrafish experiment

Tg (flk1:eGFP) transgenic zebrafish were bred and maintained in normal condition (28°C; pH 7.2‐7.4; 14 h on and 10 h off light cycles). A total of 5‐10 nL suspension containing 10 ng ctrl‐Morpholino (MO) or IMD‐MO were injected into zebrafish embryo 48 h post fertilization through the perivitelline space in a single injection by using an electronically regulated air‐pressure microinjector (Harvard Apparatus, NY, PL1‐90). For each implantation, about 50‐60 fish were transferred to six‐well plate containing 2 mL of fresh fish water. In the IMD rescue group, zIMD peptide (synthesized mature zebrafish‐IMD, VGCVLGTCQVQNLSHRLYQLVGQSGREDSPINPRSPHSY) was added into the fish water to reach a final concentration of 2 µM. The fish water was changed daily.

The living zebrafish embryos were anesthetized using 0.003% tricaine and embedded in 3% methylcellulose. The digital micrographs were acquired using Zeiss Imager Z1 fluorescence microscope (Carl Zeiss Microimaging Inc, Germany) equipped with an AxioCam MRc5 digital CCD camera or a Zeiss LSM 510 Meta Confocal Microscope. Images were taken in the same focal plane in brightfield and transmitted light passing through GFP filters (488 nm); 0.5‐2 µm step z‐stacks (512 × 512 focal planes, 50‐200 µm in depth) were acquired by using 10× or 20× objective lens. Image capture and processing were performed by using ZEISS Axiovision rel.4.8 software.

#### 4.4.1 Morpholino information

Ctrl‐MO: 5′‐CCTCTTACCTCAGTTACAATTTATA‐3′ is a standard negative control sequence, targeting a human beta‐globin intron mutation that causes beta‐thalassemia. This MO causes little change in phenotype in any known test system except human beta‐thalassemic hematopoietic cells, and been broadly used as a negative control.

IMD‐MO: 5′‐AAACCGGGAAAAGCGCTCTCATTGT‐3′ (targeting IMD or ADM2) is designed and provided by Gene Tools LLC.

### 4.5 Fibrin beads assay

Dextran‐coated Cytodex‐3 beads (GE) were coated with HUVECs at a concentration of 400 cells/bead. The following day, the HUVEC‐coated beads were washed and resuspended at a concentration of 200 beads/mL in 2.5 mg/mL of fibrinogen (Sigma) with 0.15 units/mL of aprotinin (Sigma). A total of 0.625 units of thrombin (Sigma) was added into the solution containing 500 µL fibrinogen/bead in one well of a 24‐well culture plate. Fibrinogen/bead solution was allowed to clot, and 2 × 10^4^ HSFs were plated on top of the gel clot. Medium was replaced every other day until desired growth is achieved. Reagents were added as indicated in the figure legends.

### 4.6 IP analysis

Cells were washed by ice‐cold PBS and lysed with nondenaturing lysis buffer that contains cocktail proteinase inhibitor. After centrifugation, the supernatant was extracted, and incubated with 2 µg anti‐VEC (Cat. #2500, 1:50) under continuous rotation at 4°C overnight. The mixture was then incubated with the agarose beads (Cat. D00118065, Calbiochem) under continuous rotation at 4°C for 4 h. After centrifugation, the supernatant was removed. The beads were washed by nondenaturing lysis buffer, mixed with loading buffer, and then boiled for 5 min. After centrifugation, the supernatant was collected and subjected to WB analysis.

### 4.7 WB analysis

Lysates of cells were separated by SDS‐PAGE and electrotransferred onto polyvinylidene fluoride membranes, blocked in 5% nonfat milk in Tris‐buffered saline/0.01% Tween 20 for 2 h, incubated at 4°C in Tris‐buffered saline with primary antibody (1:1000 for anti‐VEC, anti‐VEC‐Y685, anti‐VEC‐Y658, anti‐VEC‐Y731), followed by 1 h incubation with horseradish peroxidase‐conjugated secondary antibody, and detected by a chemiluminescence kit (Millipore, Cat. WBKLS0100).

### 4.8 Antibody feeding assay

Two VEC antibodies, one recognizing the extracellular domain of VEC (named as VEC‐*ext*; from BD PharMingen, Cat. 555661) and another recognizing the intracellular domain of VEC (named as VEC‐*int*; from CST, Cat. #2500), were chosen for this experiment. The HUVECs were plated on coverslips coated with 0.1% gelatine, starved overnight with 0.5% FBS, and labeled with VEC‐*ext* or nonspecific IgG for 30 min at 4°C, and stimulated with vehicle or IMD (2 µM) with or without pretreatment of SU6656 (5µM) for 20 min at 37°C. The cells were then fixed with ice‐cold acetone, permeabilized with 0.1% Triton X‐100 for 10 min, and incubated with an Alexa Fluor 488‐conjugated secondary antibody for 30 min to detect VEC‐*ext* antibodies. After successful staining was confirmed under the microscope, cells were incubated with the VEC‐*int* antibody followed by Alexa Fluor 568‐conjugated secondary antibody staining.

### 4.9 Cell surface biotinylation assay

The protocol was described previously.^28^ In brief, the cells were stimulated with IMD (2 µM) at 37°C for 10 min to induce VEC internalization. The surface receptors were then labeled with 0.5mg/mL sulfo‐NHS‐SS‐biotin (Life Technology) according to the manufacturer's instructions. After quenching the excess biotin with 100 mM glycine in PBS, the cells were dissolved in lysis buffer (25 mM Tris‐HCl at pH 7.5, 150 mM NaCl, 5 mM EDTA‐NaOH at pH 8.5, 0.5% Triton X100, 0.5% NP‐40, 100 mM NaF, 10 mM Na4 P2 O7, and 1 mM Na3 VO4) and protease inhibitor cocktail (Sigma, P2714, 1:100). The lysates were precipitated with avidin agarose beads (Life Technology) and probed for the proteins of interest.

### 4.10 Antibody feeding assay to test the interaction of internalized VEC and Rab4/Rab11

Step 1: The HUVECs were plated on coverslips coated with 0.1% gelatine, starved overnight with 0.5% FBS, and labeled with VEC‐*ext* (an antibody recognizing extracellular domain of VEC) or nonspecific IgG for 30 min at 4°C

Step 2: The labeled cells were incubated with vehicle or IMD for 20 min at 37°C to stimulate VEC internalization, and stained with an Alexa Fluor 488‐conjugated (green) secondary antibody for 30 min to detect the internalized VEC

Step 3: After successful staining was confirmed under microscope, the cells were double stained with anti‐Rab4 or anti‐Rab11 antibodies followed by Alexa Fluor 568‐conjugated (red) secondary antibody.

### 4.11 IP‐IB assay to test the interaction of phospho‐VEC and Rab4/Rab11

The HUVECs were plated on coverslips coated with 0.1% gelatine, starved overnight with 0.5% FBS, and labeled with VEC‐*ext* or nonspecific IgG for 30 min at 4°C, and stimulated with IMD for 20 min at 37°C to induce VEC phosphorylation and internalization. The cells lysates were collected and immunoprecipitated with VEC‐*ext* and VEC‐*int*, respectively. The precipitated proteins were then immunoblotted (IB) using anti‐Rab4 or anti‐Rab11antibodies. If the phosphorylated VEC preferentially binds to Rab4/Rab11, the anti‐VEC‐*ext*‐immunoprecipitated protein complex will be recognized by anti‐Rab4 and anti‐Rab11 antibodies, but the anti‐VEC‐*int*‐immunoprecipitated protein complex will not. The diagram of the experimental design is shown in Figure 6I.

### 4.12 Continuous microphotography of the living cells

HUVECs were routinely culture at 37°C and 5% CO_2_, until reached 70‐90% confluent. The lentivirus expressing mCherry‐VEC (red) was co‐transfected with the lentivirus expressing eGFP‐Rab4^WT^, eGFP‐Rab4^Act^, and eGFP‐Rab4^Neg^ or eGFP‐Rab11^WT^, eGFP‐Rab11^Act^, and eGFP‐Rab11^Neg^ (green), as indicated in the main text. The living cells were then placed under the inverted confocal microscope (Nikon TI‐DH). The double‐fluorescent images (Red/Green) were continuously acquired in a 15‐s interval, and lasted for 30‐60 min. The videos (GIF animation) were generated using the frames with a 15‐s interval. The number of long‐range moving VEC^+^, VEC^+^/Rab4^+^, or VEC^+^/Rab11^+^ vesicles in each randomly chosen field was counted. The moving distance (μm) and speed (μm/s) of the long‐range moving vesicles were also quantified. All quantifications use 10 randomly chosen fields from two independent experiments (n = 10).

### 4.13 Animal studies

All animal experiments were approved by the Animal Ethics Committee of Sichuan University and performed according to institutional and international guidelines.

#### 4.13.1 Skin transplant

WT or *IMD* ^−/−^ C57BL/6 mice (6‐8 weeks of age) were used in the skin transplantation. Mice were anesthetized using isoflurane. Hair was removed, and the surgical area was prepared with povidone‐iodine following by 70% alcohol. Full‐thickness skin was harvested from the dorsum of the donor mouse and sectioned into 1 × 1 cm grafts for subsequent transplantation. The square graft (1 × 1 cm) was placed on a graft bed prepared on the back of the recipient mouse. The graft was covered with protective bandages for 3 days. The hair regrowth in the healed graft area was considered as the skin graft regains its function.

#### 4.13.2 Tumor study

Lewis lung cancer was established in WT or *IMD* ^−/−^ C57BL/6 mice (6‐8 weeks of age) by subcutaneous injection with 2.5 × 10^6^ cells into the shaved right flank. The tumor volume was measure every 3 days after the inoculation of tumor cells. The tumor volume was determined by the following formula: Volume (mm^3^) = ½ × length (mm) × width (mm) × width (mm). Mice were anesthetized and euthanized at the end point of the tumor experiment (when the largest tumor reached about 1500 mm^3^ according to the ethical standards for animal welfare), and perfused transcardially with 2% PFA in PBS for 10 min to fix the tissues. Tumors along with adjacent normal tissues were removed, and cryosectioned at 120 µm thickness for confocal microscopy and three‐dimensional reconstruction.

#### 4.13.3 Retina study

Eyes of the neonatal WT or *IMD* ^−/−^ C57BL/6 mice (6‐8 weeks of age) were collected at postnatal day 6 (P6), and fixed with 2% PFA in PBS for 2 h. For the blood perfusion assay, the mice were anesthetized and injected with FITC‐dextran through left ventricle and allowed to circulate for 15 min for staining the perfused vessels. Retinas were carefully dissected, washed with PBS, and blocked with 10% goat serum in PBST for 3 h, and then incubated overnight with AlexaFluor568‐conjugated IB4 to label the whole retinal vasculature.

### 4.14 Acquisition of microscopic images

The microscopic images from the Fibrin beads assay were acquired using Nikon TE2000 inverted microscope. Images of tissue slides, including the normal dermis, wound healing, and tumor tissues, were acquired using Nikon A1RMP+ upright confocal microscope. Images of a whole retinal vasculature were acquired using Zeiss Z2 upright fluorescence microscope and automatically combined with 25 continuous shooting images by the software Axiovision. Images of cell slides, including the immunostaining of VEC, Rab4, Rab11, and Phalloidin, were acquired using Zeiss Z2 upright fluorescence microscope. The continuous microphotography (to observe the movement of VEC^+^/Rab4 or VEC^+^/Rab11^+^ vesicles in living cells) was performed using Nikon TI‐DH inverted confocal microscope with a 15‐s interval.

### 4.15 Fluorescent immunostaining and quantification

Tissue/cell slides were fixed with 4% PFA and stained with anti‐VEC (Cat. #2500 or Cat. 555661, 1:100), anti‐CD31 (1:50), anti‐IMD (1:100), anti‐Rab4 (1:100), anti‐Rab11 (1:100), or Alexa Fluor 568‐conjugated phalloidin (1 µg/mL) and DAPI (20 µg/mL), followed by staining with Alexa Fluor 488‐ or 568‐conjugated secondary antibody (1:200).

The staining density of VEC relative to phalloidin (Figure 3F) was quantified using Image‐Pro Plus v5.0.2.9. The quantification method was as follows: Step 1, the area of cell‐cell contacts was selected, and the intensity of F‐actin (phalloidin‐positive) positive signal was quantified; Step 2, the intensity of VEC signal in the same area was quantified; Step 3, the ratio of the intensities of VEC/phalloidin staining in one field was calculated and expressed as a dot in the statistical graph. The relative intensity of the VEC signal to phalloidin signal at cell‐cell contacts was quantified from 10 randomly chosen fields in two experiments.

### 4.16 Statistics

When comparing two groups for which a Gaussian distribution was not assumed, the unpaired, two‐tailed nonparametric Mann‐Whitney *U* test was used; when a Gaussian distribution was assumed, the unpaired, two‐tailed parametric *t* test with Welch's correction was used. Data from multiple groups were compared using one‐way ANOVA (Kruskal‐Wallis test) followed by nonparametric Dunn's post hoc analysis. A *P* value < .05 was considered statistically significant. *P < .05, **P < .01, and ***P < .001. The n‐number of each experiment was indicated in figures. In animal studies, no randomization was applied because all mice used were genetically defined, inbred mice.

## Supporting information

Supporting Information

Supporting Video S1

Supporting Video S2

Supporting Video S3

Supporting Video S4

Supporting Video S5

Supporting Video S6

Supporting Video S7

Supporting Video S8
