# Supplementary material for: Intermedin promotes vessel fusion by inducing VE‐cadherin accumulation at potential fusion sites and to achieve a dynamic balance between VE‐cadherin‐complex dissociation/reconstitution
Source: MedComm (2020). 2020 Jun 9;1(1):84–102. doi: 10.1002/mco2.9 (PMC8489673; doi:10.1002/mco2.9)
Supplement: Supplementary file 1 — Supporting Information [file MCO2-1-84-s009.docx]

**
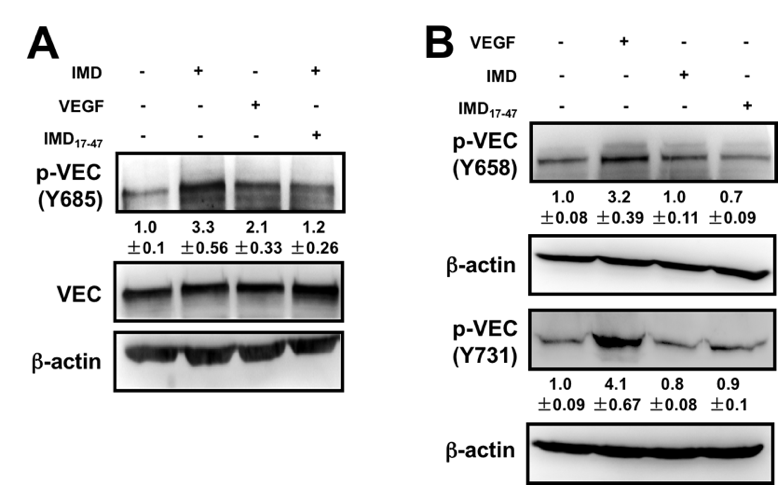
**

**Supplementary Figure 1. IMD induced VEC phosphorylation at Y685, not Y731 or Y658.** Samples from HUVECs treated by IMD, VEGF, or IMD inhibitor (IMD^17-47^) were immunoblotted by anti-pY685-VEC (**A**), or anti-pY658-VEC/anti-pY731-VEC (**B**). The level of p-VEC (referred to t-VEC) was presented relative to control; n=3.

**
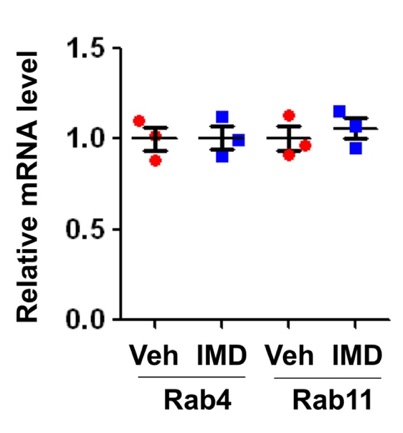
**

**Supplementary Figure 2. IMD did not affect the mRNA level of Rab4 or Rab11.** The HUVECs were treated with or without IMD. The mRNA level of Rab4 or Rab11 was measured by Real-time RT-PCR.

**
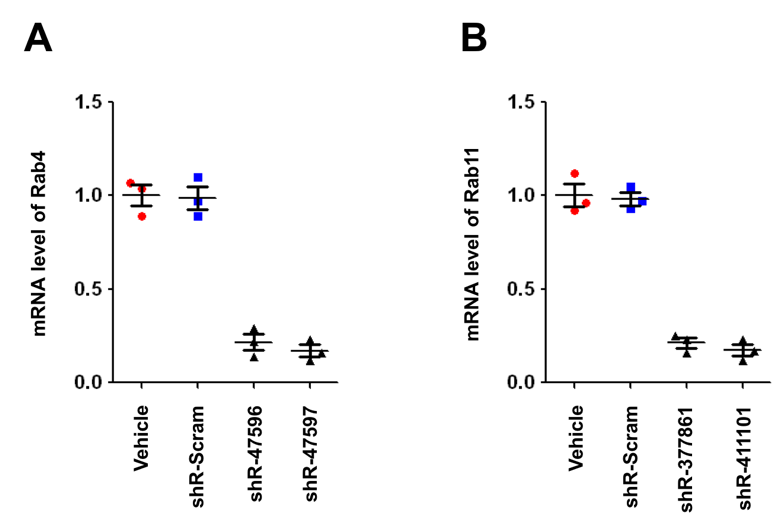
**

**Supplementary Figure 3. The knockdown of Rab4 or Rab11.** The HUVECs were transfected with the shRNA candidates of Rab4 (**a**) or Rab11 (**b**). The mRNA level of Rab4 or Rab11 was measured by Real-time RT-PCR.
